# Minor Thyroid Insufficiency

**Published:** 1919-05-10

**Authors:** 


### MINOR THYROID INSUFFICIENCY.

### A Condition Met With Daily.

From amongst the less well-favoured of our fellow travellers in the streets and trains, many can be selected who, although themselves unconscious of any departure from the normal, have suffered some common type of misfortune. One such class is that which shows the effects of inadequate secretion of the thyroid gland. Its numerous members are known by the premature greyness and scantiness of their hair, which recedes both from the occiput and forehead, by the thinness of the outer half of each eyebrow, by a certain coarseness of feature and a tendency to puffiness under the eyes, by the harshness of their skin and the brittleness of their nails, by their obvious chilliness in winter, by the blueness of their hands and ears, and an unnatural flush over their malar bones. The grosser forms of this disorder are easy to detect, but for the diagnosis of the mild degrees practice is required, and an aptitude to see our fellow creatures as they ought to be rather than as they are.

The thyroid regulates growth, and various degrees of dwarfism follow upon its defective action in early life. The minor failures of development alone concern us here. They may be of a cretinoid type, with definite stigmata, or of the Lorain type, in which the evolution of the various parts is proportionate, but the whole somewhat diminutive. The epiphyses are late in uniting, and, until their union with the bony shafts is complete, increase in stature is possible under appropriate treatment. A series of radiograms should always be examined before a prognosis is made regarding the possibility of growth.

Thyroid secretion also regulates katabolism, and when insufficient the tissues become clogged with a mucin-like substance which is probably an intermediate product of katabolism.

These two factors—namely, diminished rate of combustion and myxoedematous infiltration—account for many of the signs and symptoms of the disease. Internal temperature is subnormal; there is a subjective sensation of cold, particularly in the hands and feet, and a tendency to shiver even when heavily clothed. Winter is a time of great discomfort, and unless there is coincident visceroptosis a decided preference is evinced for hot weather.

Infiltration of the subcutaneous tissues is not pronounced in the mild varieties under discussion, and gives rise merely to a thickening of the features and a full habit of body, the latter by no means always conspicuous. Infiltration of the muscles and of the tissues around the joints often causes pain and stiffness of movement, producing a condition which is frequently mistaken for muscular rheumatism or even rheumatoid arthritis. The pains, which are usually worse at night, affect all parts. Most frequently, however, they are felt in the dorsal musculature, where they constitute an intractable backache.

In the digestive system peristalsis becomes sluggish, and the abdominal musculature so weakened that constipation and visceroptosis develop. Appetite fails and is often replaced by distaste for food, especially for meat. Secondary changes affect all parts of the alimentary tract, and manifest themselves as oral sepsis, various forms of indigestion, and of abdominal pain, the prominence of which symptoms may so compel the attention that the underlying subthyroidism is overlooked.

In certain cases the sensitiveness of the bladder increases, possibly because of a myxoedematous infiltration of the trigone, and a constant desire to micturate is experienced. Nocturnal enuresis is also common, and the possibility of subthyroidism should always be borne in mind when one is confronted with this disorder.

The thyroid gland has a profound influence over the organs of sex, not only regulating their development, but also assisting in the maintenance of their functional activity. In the subthyroidic the uterus, ovaries, and testicles develop late, and the suprapubic hair is scanty. Menstruation is delayed in its onset and abnormal in character, an excessive amount of blood usually being lost. Fertilisation of the ovum is just as likely to occur in a subthyroidic woman as in a normal one, but the embryo is often carried away by a profuse flow at a later menstrual epoch. During pregnancy the activity of the thyroid gland increases, and subthyroidic women often feel fitter during this period, although their symptoms begin to return after the puerperium. Usually lactation is imperfect, and menstruation recommences quickly.

Nerve and mental symptoms are numerous and of very great importance. Sensations of tingling and of "pins and needles" are commonly experienced in the limbs. Headache on waking is very characteristic and may resemble that of frontal sinus catarrh or radiate upwards from the occiput after the fashion of neuralgia. Like so many of the symptoms of benign subthyroidism, these headaches improve towards evening, especially after a good dinner with wine. Noises in the ears and vertigo are also common. The latter may occasion a fall, although in its slighter forms it merely gives rise to a sense of insecurity. General mental torpidity, impairment of memory, defective powers of association, and a difficulty in verbal expression are extremely common. Somnolence and heaviness of sleep are almost invariably present. The outlook on life is usually one of depression and melancholy.

The disease attains a maximum incidence amongst the middle-aged women of the so-called working-classes, and is the cause of much misery in the homes of the poor. The afflicted one can only discharge her responsibilities imperfectly and is unable to maintain the efficiency of her household. Ill-planned expenditure and effort, dirtiness and disorder, combined with the patient's own physical

**Minor Thyroid Insufficiency—(*continued*).**

sufferings and the psychic atmosphere of irritable, suspicious pessimism which emanates from her, produce a condition of distress only rivalled by that of homes which are under the ban of drunkenness.

Diagnosis, therefore, becomes a social obligation and treatment quickly brings its recompense. The subthyroidic do not tolerate thyroid extract so readily as do the healthy, so that the initial dose should be small and systematic increases made at short intervals. The tachycardia and palpitation which often follow act as warnings that the dose must only be increased with caution, but are not signs of hyperthyroidism. The heart muscle is impaired in vitality and shares the general myxoedematous infiltration. Its mechanical efficiency temporarily is lessened by the removal of the myxoedematous padding at a time when a general rise in metabolic activity is calling for a vigorous cardiac response. Consequently symptoms of cardiovascular distress are experienced. In severe cases the patient should be kept in bed for a short period when treatment is first started so that much of the strain being removed the dose can be increased more quickly.

Too rapid depletion may produce untoward effects in other parts. Undue laxity of ligaments is sometimes induced with resultant instability and deformities, one of the commonest of which is knock-knee. Any pre-existing tendency to visceroptosis is aggravated. The loss of hair which frequently occurs at first is merely a loss of an imperfect growth. Reassurance can be given with confidence that a more luxuriant crop of more lustrous hair will soon replace the old.

The effects of correct dosage are prompt and dramatic. The rate of metabolism increases and the patient's weight falls steadily. The output of carbon-dioxide from the lungs and of urea from the kidneys rise to a much higher level than that of health. This phase, however, ends when the combustion of the myxoedematous store is complete. Metabolism then returns to a normal rate and weight remains constant. After a varying interval the weight often rises again even in adults. This secondary gain is due to the development of healthy tissues and to a general improvement in nutrition. It must not be confused with the relapse which ensues if the patient becomes careless and omits to take thyroid extract. When this stage has been reached the most conspicuous part of the treatment has been accomplished, but much still remains to be done. Many cases of subthyroidism are due to antenatal toxaemias, such as those of tuberculous and syphilitic infection in the parents. The gland having been damaged before birth is beyond all therapeutic aid, although its lesion is non-progressive. Many glands, however, are injured after birth, sometimes by acute and transient causes such as the exanthemata, but very frequently by chronic and persistent causes which produce slowly increasing lesions. Such harmful agents are chronic alcoholism, chronic lead-poisoning, the chronic bacteriaemias and toxaemias which arise from oral sepsis, chronic throat infections and disorders of the lower parts of the digestive tract, chronic exhaustion from overwork, excessive child-bearing and incessant worry. The detection and eradication of these deleterious factors call for a careful clinical and sociological survey. Their control prevents further destruction of the thyroid gland and may even permit of its partial regeneration.

A general plan of treatment is to give half a grain of standardised thyroid extract nightly for about two weeks, and then give a second daily dose in the mornings. A little later the extract may be given three times a day, and in the class of benign myxoedema under discussion it is rarely necessary to increase the dose beyond this point. As a rule better results are attained by supplementing the thyroid extract with an adjuvant drug such as grey powder and soda, or arsenic iodide or calcium iodide, but these drugs should never be given for long periods at a time.

All ætiological factors capable of increasing the defect in the gland should then, as far as possible, be controlled, remembering that amongst these are included not only the original causes, but also chronic infections which have arisen in consequence of the subthyroidic state.

Finally, the minimum dose of thyroid extract necessary to maintain the efficiency which has been established should be ascertained by careful trial and this dose prescribed for life-long usage.

Examinations from time to time are advisable for the detection of any tendency to relapse, induced by uncontrolled factors which have increased the lesion in the gland and called for renewed efforts and an increased dosage.

